# Early pathways of maternal mentalization: Associations with child development in the FinnBrain birth cohort study

**DOI:** 10.3389/fpsyg.2022.855190

**Published:** 2022-12-05

**Authors:** J. Lindblom, M. Pajulo, S. Nolvi, K. Tervahartiala, H. Karlsson, L. Karlsson, R. Korja

**Affiliations:** ^1^The FinnBrain Birth Cohort Study, Turku Brain and Mind Center, Department of Clinical Medicine, University of Turku, Turku, Finland; ^2^Faculty of Social Sciences, Tampere University, Tampere, Finland; ^3^Department of Child Psychiatry, University of Turku and Turku University Hospital, Turku, Finland; ^4^Turku Institute for Advanced Studies, University of Turku, Turku, Finland; ^5^Department of Psychology and Speech Language Pathology, University of Turku, Turku, Finland; ^6^Centre for Population Health Research, University of Turku and Turku University, Turku, Finland; ^7^Department of Psychiatry, Turku University Hospital and University of Turku, Turku, Finland

**Keywords:** parental mentalization, reflective function, perinatal, postnatal, infancy, child development, trajectories, longitudinal

## Abstract

Parental mentalization refers to a parents’ capacity and interest to consider the individual experience and mental state underlying the behaviors of the child. Higher mentalization is considered a key aspect for parental sensitivity in interaction, fostering child’s socioemotional and self-regulatory development. Yet, previous studies have not examined the dynamic pathways through which the maternal mentalization may develop, nor their effects on child development. Thus, in the current person-oriented study, first, we identify distinct profiles and longitudinal trajectories of maternal mentalization from pregnancy to child’s 2 years of age. Second, we test how the profiles and trajectories associate with children’s internalizing and externalizing problems, social–emotional competence and effortful control at the age of 2 years. Third, we examine how the profiles and trajectories associate with contextual demographic and child related. The substudy was part of the FinnBrain Birth Cohort and included families from general population (*n* = 2,687). Mothers reported their parental reflective functioning (PRF) at late pregnancy, 6 months and 2 years of child’s age. Both mothers (*n* = 1,437) and fathers (*n* = 715) reported the developmental child outcomes at the child’s age of 2 years. Latent Profile Analysis and Latent Transition Analysis were used to identify PRF profiles and trajectories. The results showed decreasing heterogeneity in PRF from pregnancy to child’s age of 6 months and 2 years (i.e., four, three and two latent classes, respectively). Most mothers progressed towards high PRF over time. Second, the profiles and trajectories depicting high PRF associated with child high social–emotional competence at the age of 2 years, yet no clear positive effects were found on child’s problems and effortful control. The group of mixed PRF trajectories showed strongest association with child’s internalizing and externalizing problems. Finally, there were theoretically meaningful associations between the PRF trajectories and both the contextual (e.g., parity) and child related (e.g., infant temperament) factors. This was the first study to explore the early unfolding of maternal mentalization. The results are discussed in relation with the potential mechanisms accounting for child development and with the nature and limitations of self-reported parental mentalization.

## Introduction

Parental mentalization has been suggested to be critically important for the child’s early social, emotional, and cognitive development (Sharp and Fonagy, 2008). It refers to a parent’s effort to make sense of her/his child as a separate individual person with his/her own thoughts, feelings, and mind. The development of parental mentalization starts already during pregnancy, when it expresses itself as the parent’s orientation to the experiences of the fetus-baby and making room for the child both in mind and practice (Slade, 2002). After child’s birth, parental mentalization is considered a pre-requisite for sensitive caregiving, allowing the parent to respond appropriately to child’s subtle cues and provide an essential feedback on child’s internal states (Provenzi et al., 2018). Such processes are assumed to be beneficial on the child’s early development of self-regulation and social competence (Fonagy, 2006; Sharp and Fonagy, 2008). In contrast, problems in parental mentalization is considered detrimental and it can heighten the risk for various forms of child’s socio-emotional and behavioral problems (Fonagy et al., 2002).

Studies on the development of mentalization in early parenthood are scarce. While many studies have assessed maternal mentalization on single occasions (e.g., Salo et al., 2021), studies with repeated assessments are needed to study how the parental mentalization develops over time. Therefore, in the current longitudinal study, we chart the pathways through which maternal mentalization change and evolve from pregnancy to infancy and toddlerhood. To achieve this, we utilize the recently developed mentalization questionnaires applicable during both pregnancy and after the child’s birth. Moreover, we utilize person-oriented methodology to identify distinct mentalization profiles and longitudinal trajectories (Bergman and Trost, 2006). The benefit of this data-driven approach is that it allows the identification of subgroups of mothers with similar mentalization patterns. Finally, we test how the maternal mentalization profiles and trajectories associate with child socio-emotional development and self-regulation.

Mentalization refers to a person’s capacity and willingness to consider oneself and other people in terms of mental states; that is, in terms of feelings, beliefs, intentions and desires. It is an effort to think of experience behind overt behavior (Fonagy et al., 2002). “Reflective functioning” (RF), an operationalized term for mentalization, refers to the measured degree and aspects of mentalization. RF has been presented to occur along a continuum, from absence or denial of mental states at one extreme, to exceptionally rich understanding of both the nature and dynamic interplay of mental states within and between people (Fonagy et al., 2002, 2012). The theory of mentalization is closely connected with developmental research on “a theory of mind,” that is, understanding that all people have their own and separate minds and perspectives to situations and things (Baron-Cohen, 1995).

Parental reflective functioning (PRF; Slade, 2005; Slade et al., 2005), in specific, refers to a parent’s RF in relation to one’s own child. It includes cognitive aspects like psychological insight and perspective taking, but also emotional aspects like capacity to fully experience the child’s emotions without becoming overwhelmed oneself (Slade, 2005). A parent with good PRF gives value to thinking of the child’s experience but also acknowledges the impossibility to know it for certain (Slade et al., 2009; Pajulo et al., 2015).

The task to mentalize is the more challenging the younger the child is (Slade et al., 2010). When the child is not yet born, the task is qualitatively different compared to the later postnatal period (Slade, 2011). During pregnancy high PRF includes the parent’s ability to reflect and integrate on mental states in own past, current and future relationships. Prenatal PRF also manifests as parent’s curiosity and capacity to think of the fetus-child as a separate individual, with own developing personal features and needs. It includes parent’s interest and curiosity towards the fetus-baby’s developing capacities and experience, and willingness to think of the reactions of the baby in relation to the parent’s own actions and mental states (Slade, 2002, 2011; Pajulo et al., 2006, 2016, 2015).

According to the current views, PRF is a multidimensional process. The Parental Reflective Functioning Questionnaire (PRFQ), a self-report measure of PRF, has been confirmed to depict three dimensions among parents with young children (Luyten et al., 2017; Wendelboe et al., 2021). The first dimension, *certainty of mental states (“Certainty”)*, ranges from the parent being extremely certain about the mental states of the child to an almost complete lack of confidence about child’s mental states. The second dimension, *interest and curiosity (“Interest”)* in the child’s mental states, refers to the parent’s willingness and interest toward the child’s experience, perspective and mind (Njissens et al., 2021). Very high scores on these dimensions have been suggested to indicate maladaptive hypermentalizing, involving over-interpretating or “knowing” what their child feels or thinks, while medium range scores (especially on Certainty) have been suggested to indicate more adaptive mentalization (Fonagy and Luyten, (2009); Pajulo et al., 2015). However, empirical evidence for the curvilinear effects of these dimensions has been equivocal, many findings suggesting that high scores associate with more adaptive parenting (Luyten et al., 2017). The third dimension, *prementalizing modes (“Prementalizing”)*, refers to parents’ tendency to make malevolent attributions about the child. While it clearly refers to impaired PRF, it has shown somewhat limited psychometric properties (Luyten et al., 2017; Wendelboe et al., 2021), perhaps due to relative rare occurrence of severe prementalizing. During pregnancy, the PRF involves an additional dimension regarding *dynamics of mental states between different relationships (“Dynamics”)*, that characterizes parent’s ability and willingness to consider one’s own past, current and future relationships (Pajulo et al., 2015; Vahidi et al., 2021).

After the child is born, PRF is considered to be influential on child development largely through parental sensitivity in parent–child interactions (Fonagy et al., 2002; Slade, 2005; Sharp and Fonagy, 2008). Parental sensitivity refers to parent acknowledging the child’s cues, interpreting, and responding to them timely, accurately, and often enough (Ainsworth et al., 1978). Thus, a parent with high PRF is more flexible and open to think alternative explanations underlying the child’s overt reactions and behavior, and has hence better chances to respond sensitively (Slade, 2005; Slade et al., 2005). High PRF also allows the parent to reflect on feelings in conflictual situations and thus foster the capacity to regulate emotions in interaction, both own and the child’s (Lyons-Ruth, 1999). Accordingly, when the parental mentalization is inept, for whatever reason, it can lead to weakened own mentalization and regulatory capacity in the child, and *via* those, to the development of emotional, behavioral and social problems (Sharp and Fonagy, 2008).

Considerable amount of empirical evidence is available about the role of PRF on parenting as well as on child development. For example, Camoirano (2017) reviewed 47 studies that have utilized various interview methods to assess PRF. Taken together, the reviewed studies supported the notion that higher PRF associates with higher maternal sensitivity and higher quality of parenting, as well as with mothers efficient emotion regulation. Moreover, in a meta-analysis of 20 studies higher maternal PRF was found to associate with infants’ higher attachment security and children’s own mentalizing abilities (Zeegers et al., 2017). There is also evidence that parenting practices reflecting high mentalization associate with child’s lower externalizing and internalizing problems (Camoirano, 2017), and better self-regulation and social cognition (Camoirano, 2017; Aldrich et al., 2021).

Research is still limited on the self-report questionnaire measures of PRF, regarding associations with parenting and child development. Luyten et al. (2017) found that the dimensions of PRFQ (i.e., Certainty, Interest and Prementalizing) associated with self-reported emotional availability in mothers of one-year-old children. Similarly, De Roo et al. (2019) found associations between high Interest and Curiosity and self-reported better parental coping and self-efficacy among mothers of children under the age of 12 years. Rutherford et al. (2013, 2015) found high Interest to predict mother’s tolerance of infant distress in a simulated stress provoking caregiving situation. Rostad and Whitaker (2016) found that parents with higher Interest and Certainty had greater parental involvement in their self-reported parenting, and Certainty associated with higher self-reported limit setting and more effective communication with the child. Recently, in a study with a large sample of general population first-time parents, Salo et al. (2021) found that parental PRF (Interest and Prementalizing combined) mediated the link from marital satisfaction to emotional availability in mother–child interaction, as well to child’s behavioral and emotional problems, but not to social competence, at the age of 12 months. Altogether, while these studies provide evidence for the validity of the PRFQ, research is scarce on child outcomes. Furthermore, previous studies have rarely utilized longitudinal designs. Thus, very little is yet known about the longitudinal course of maternal mentalization and their associations with child development.

Most previous studies have assessed PRF using interview methods, and there is still rather scarce number of studies that have used the parental self-report questionnaire measures. Despite their obvious limitations, self-report questionnaires enable larger study samples and open new avenues to study mentalization. The main aim of this longitudinal study is to examine how maternal mentalization, as assessed with PRFQ, changes and evolves from pregnancy to child’s infancy and toddlerhood in a general population sample. Methodologically, we utilize person-oriented approach, that aims to statistically identify homogenous subgroups of individuals based on patterns across multiple variables (Bergman and Trost, 2006). In other words, our aim is to identify and describe naturally occurring groups of mothers with distinct PRF profiles and longitudinal trajectories. The approach stands in contrast to the more common variable-oriented approach that tends to focus on the whole-sample average level effects or to utilize some pre-defined subgroups (e.g., diagnostic groups). In addition, the study adds knowledge to previous literature in terms of exploring how maternal PRF trajectories associate with child outcomes. We focus on child’s internalizing and externalizing problems, social–emotional competence, and self-regulation, as the theoretically salient developmental outcomes (Camoirano, 2017; Aldrich et al., 2021).

We deem it noteworthy to acknowledge the importance of multiple contextual factors that can shape both parenting and child development (Sameroff, 2010). For example, higher parental education has been found to associate with parenting in complex ways (Ensminger and Fitherhill, 2003). Regarding PRF, higher parental education level has been reported to associate with high PRF (Salo et al., 2021). Furthermore, the experience of transitioning to parenthood is often especially intense for the first-time mothers. Indeed, some studies indicate higher PRF (Pajulo et al., 2015; Salo et al., 2021) and higher maternal-fetal attachment (McNamara et al., 2019) among primiparous compared to multiparous mothers. Finally, while some studies suggest that older mothers are psychologically more resilient in the early parenthood (McMahon et al., 2011), older mothers also tend to experience lower maternal-fetal attachment during pregnancy (McNamara et al., 2019).

In addition, child related factors can be highly influential on the child development and parent–child relationship (Sameroff, 2010). Biologically based temperament is known to shape child’s emotional, social and regulatory development (Rothbart and Bates, 2006). For example, infant’s high negative affectivity and lower regulating/orienting has been found to associate with heightened internalizing and externalizing symptoms, as well as social problems, at the age of 2 years (Peterson et al., 2018). Moreover, boys tend to show lower self-regulation than girls, in terms of both lower effortful control (e.g., inhibitory and attention control) and higher incidence of externalizing problems (Else-Quest et al., 2006). Interestingly, the child related factors can also influence parenting by evoking differential caregiving responses (Kiff et al., 2011; Wittig and Rodriguez, 2019). For example, infant’s negative affectivity tends to evoke more controlling forms of parenting and less parental affection (Kiff et al., 2011). To understand the formation of early maternal PRF and its associations with child development, it is important consider the roles of both demographic and child related factors.

In the current study, our *first aim* is to explore how maternal PRF changes and evolves from pregnancy to child’s age of 6 months and 2 years. Using person-oriented methods, we identify PRF profiles (i.e., latent classes) separately at the three time points, and model transitions in mother’s PRF that occur across time from pregnancy to child’s toddlerhood (i.e., trajectories). Identification of the profiles is based on three PRF dimensions during pregnancy (i.e., certainty, interest, and dynamics) and two PRF dimensions after child’s birth (i.e., Certainty and Interest). As this is the first person-oriented study on PRF, we did not form specific hypotheses about the content of the profiles or trajectories. Yet, we expected them to reflect the typical positive progression of maternal PRF that has been observed in previous variable-oriented studies during this phase (e.g., mostly transitions from lower to higher PRF).

Our *second aim* is to test how the identified maternal PRF profiles and trajectories associate with different aspects of child outcomes at the age of 2 years, including child’s internalizing and externalizing problems, social–emotional competence, and effortful control. We expected trajectories characterized by high stable maternal PRF (compared to trajectories characterized by lower PRF) to associate with child’s lower externalizing and internalizing problems, higher social–emotional competence and higher effortful control at the child’s age of 2 years. To gain more objective and comprehensive estimation of the child outcomes, we combined maternal and paternal reports.

Our *third aim* is to explore the associations between the contextual demographic and child related factors with the maternal PRF profiles and trajectories. As the demographic factors, we focused on maternal age, education level, and parity status. Regarding the child related factors we focused on child’s gender and infant temperament (i.e., negative affectivity, surgency and regulation/orienting) at the age of 6 months. Due to lack of previous research, we did not form specific hypotheses about the associations with the PRF profiles and trajectories.

## Materials and methods

### Participants and study design

The current study is part of the FinnBrain Birth Cohort Study^1^ (*n =* 3,808 familes). FinnBrain is a multidisciplinary population-based longitudinal study exploring different risk and resilient factors for child development. The participants were recruited from maternal welfare clinics at 12 weeks of pregnancy in the city of Turku in Southwest Finland, municipalities in the Turku area and the Åland Islands. According to the study inclusion criteria, the nurses recruited families with sufficient knowledge of Finnish or Swedish and selected children with a normal fetal ultrasound screening status (Karlsson et al., 2018). The current substudy included *n =* 2,687 families, with reports from mothers (*n =* 2,578) during pregnancy at 32 gestational weeks (T1), reports from mothers (*n =* 1916) and fathers (*n =* 1,020) at the child’s age of 6 months (T2), and reports from mothers (*n =* 1,444) and fathers (*n =* 723) at the child’s age of 2 year (T3). Mothers’ reports included maternal PRF (T1-T3), infant temperament (T2) and child outcomes (T3), and fathers’ reports regarded infant temperament (T2) and child outcomes (T3). Written informed consent was obtained from the parents. The ethical committee of South-West Hospital District of Finland has approved the study protocol and the study was conducted in compliance with the Declaration of Helsinki.

### Measures of parental reflective functioning

#### Prenatal parental reflective functioning at T1

Maternal mentalization during pregnancy was assessed using Prenatal Parental Reflective Functioning Questionnaire (P-PRFQ) (Pajulo et al., 2015). It consists of 14-item that are answered using a Likert-scale (1 = *“strongly disagree,”* 7 = *“strongly agree”*). The items assess three PRF subscales: Certainty (4 items; α = 0.76) assesses the extent to which a parent is (or is not) aware of the opacity of mental states and limitations in knowing them for certain, especially so regarding unborn babies and small children (e.g., “*As a parent, I think I will always know why my child acts the way that she/he does*”). Interest (5 items; α = 0.74) refers to a parent being willing and curious to consider the baby’s perspective, experience and needs already during pregnancy (e.g., *“I find it fascinating to search for signs that would tell me how my developing baby is doing*.*”*). Dynamics (5 items; α = 0.71) refers to a parent’s ability to consider the dynamic nature of mental states over time and integrating one’s own past relationship with own parent to the present time of pregnancy, and towards future relationship with this particular baby (e.g., *“Nowadays I find myself thinking of how it may have been for my mother when she was pregnant with me”*). P-PRFQ has been validated against the Pregnancy Interview method by Slade (2011) in Pajulo et al. (2015). In the current study the items were scored to accord with the postnatal assessment (Luyten et al., 2017), that is, higher scores presenting higher certainty, interest or dynamics. The used subscales and items are presented in Supplementary Material S1.

#### Postnatal parental reflective functioning at T2 and at T3

Maternal mentalization was assessed using Parental Reflective Functioning Questionnaire (PRFQ) (Luyten et al., 2017) at the child’s age of 6 months (T2) and 2 years (T3). Because the PRFQ was used in an early postnatal phase (T2) for the first time in the current birth cohort study, some modifications on wording were done to the questionnaire like using “baby” instead of “child,” and “reaction” instead of “behavior” for version used at T2 (for details, see PRFQ-Fi) (Pajulo et al., 2018). Furthermore, based on pilot testing (Pajulo et al., 2018), two items from Prementalizing scale at T2 were excluded as the mothers of young infants experiences these confusing and not applicable (i.e., “*I find it hard to actively participate in make-believe play with my child”* and *“When my child is fussy, he or she does that just to annoy me”*). At T3 the original phrasing of PRFQ was used. Consequently, at T2, the PRFQ included 16 items, and at T3, the PRFQ included 18 items, all answered using a Likert-scale (1 = *“Strongly disagree,”* 7 = *“Strongly agree”*).

The PRFQ items assess three mentalization subscales: Certainty (6 items; T2: α = 0.80, T3: α = 0.81) refers the extent to which a parent is (or is not) aware of the limitations and opacity in knowing mental states for certain (e.g., *“I always know why my child acts the way he or she does”*). Interest (6 items; T2: α = 0.81, T3: α = 0.80) refers to the parent being curious to acknowledge and consider the baby’s perspective, experience and needs (e.g., *“I am often curious to find out how my child feels”*). Prementalizing (T2: 4 items, α = 0.41, T3: 6 items, α = 0.47) refers to parent’s tendency to make maladaptive, inappropriate or malevolent attributions about the child (e.g., *“My child cries around strangers to embarrass me”*). Unfortunately, considering the unsatisfactory reliability (well below the recommended α ≥ 0.70; Taber (2018)) and the complexity of our statistical analyses, we decided to exclude Prementalizing from further analyses. The used items in the current study at T2 and T3 were scored following Luyten et al. (2017), that is, higher scores presenting higher certainty or interest. The used subscales and items are presented in Supplementary Material S2.

### Measures of child outcomes

#### Child’s problems and social–emotional competence at T3

Child’s problem and social–emotional competencies were assessed using Brief Infant-Toddler Social Emotional Assessment (BITSEA) at the child’s age of 2 years (Briggs-gowan et al., 2002). The BITSEA is a brief comprehensive screening instrument designed to evaluate social and emotional behavior in small children aged 12–35 months. Both mothers and fathers were asked to fill the questionnaire. In the current study, we used three subscales: Externalizing problems (6 items, mothers α = 0.61, fathers α = 0.61) covering aggression, defiance, and activity/impulsivity items. Internalizing problems (8 items; mothers α = 0.59, fathers α = 0.59) covering depression, anxiety, and negative emotionality. Competence (11 items, mothers α = 0.59, fathers α = 0.62) covering prosocial peer relations, empathy, imitation/play skills, social relatedness, attention, compliance, and mastery motivation. Responses from mothers and fathers correlated with each other, *r*’s ranging from 0.33 to 0.44, all *p’s* < 0.001, and were averaged together.

#### Child’s effortful control at T3

Child’s effortful control was assessed using The Early Childhood Behavior Questionnaire (ECBQ) at 2 years (Putnam et al., 2006, 2014). The ECBQ is a parental self-report designed to measure temperament in toddler-aged children based on the child behavior during the past 2 weeks, answered using a Likert scale (1 = “Never,” 7 = “All the time”). To measure child’s self-regulation, we utilized only the effortful control subscale (32 items; mothers α = 0.86, fathers α = 0.86). The items cover domains of inhibitory control, attentional shifting, low intensity pleasure, cuddliness and attentional focusing. Responses from mothers and fathers correlated with each other, *r* = 0.30, *p* < 0.001, and were averaged together.

### Measures of demographic and child related factors

#### Demographic variables

As covariates in the analyses, we used maternal age (in years), mother’s education level (1 = High school/vocational education, 2 = Applied university, 3 = University degree), and parity status (0 = primiparity, 1 = non-primiparity) and child’s gender (0 = girl, 1 = boy). Maternal age and education were gathered at the baseline (gestational week 14).

#### Infant temperament at T2

Infant temperament was assessed using The Infant Behavior Questionnaire Revised (IBQ-R) (Gartstein and Rothbart, 2003). Both mothers and fathers were asked to rate their infants’ observed behavior during the past weeks using a Likert scale (1 = *“never,”* 7 = *“always”*). The questionnaire contains three broad dimensions: Negative affectivity (10 items; mothers α = 0.85, fathers α = 0.86) covering distress to limitations, fearfulness, sadness and recovery from negative emotions (reverse scored); Surgency (10 items; mothers α = 0.88, fathers α = 0.89), covering activity level, smiling and laughter, high intensity pleasure, perceptual sensitivity, approach and vocal reactivity; and Regulation/orienting (10 items; mothers α = 0.83, fathers α = 0.85) covering low intensity pleasure, cuddliness, duration of orienting, and soothability. Responses from mothers and fathers correlated with each other (*r*’s ranging from 0.34 to 0.49, all *p’s* < 0.001) and were averaged together.

### Statistical methods and analyses

The main analyses were conducted using Mplus 8.5 (Muthén and Muthén, 1998) and SPSS version 27. To account missingness and non-normality of the variables maximum likelihood (FIML) estimation with robust standard errors was used in all Mplus analyses. To depict the PRF profiles and trajectories, we conducted a Latent Transition Analysis (LTA) (Collins and Lanza, 2009). It is a data-driven and person-oriented method that identifies categories of individuals with similar multivariate profiles (referred to as latent classes). Moreover, it can be used to depict how the individuals move (or stay) between the latent classes over time, even if different classes have emerged at different timepoints. In the first phase of the analysis a Latent Profile Analysis (LPA) is conducted to identify latent classes cross-sectionally at each time point. In the second phase of the analyses the probabilities of transitioning from each latent class at one time point to all others at the next time point are estimated.

In the first phase of LTA, we ran the LPA separately using maternal PRF variables assessed either at pregnancy (T1), at child’s age of 6 months (T2), or at child’s age of 2 years (T3). The latent classes were based on participants similarity in the means of the indicator variables (three at T1, and two at T2 and T3). The optimal number of the latent classes was based on multiple criteria, involving Akaike’s Information Criterion (AIC; Akaike, 1987), Bayesian Information Criterion (BIC; Schwarz, 1978), and the adjusted BIC (aBIC; Sclove, 1987). In addition, we used the Bootstrapped Log Likelihood Ratio Test (BLRT; McLachlan and Peel, 2000) and the Vuong-Lo–Mendell–Rubin Test (VLMR; Vuong, 1989; Lo et al., 2001) to tests for the optimal number of classes. Entropy statistic was used to describe the clarity of the selected solution (Celeux and Soromenho, 1996).

In the second phase of the LTA, after identifying the latent classes cross-sectionally we combined the classes and estimated the probabilities of change over time from one latent class to another. Change over time is represented by the probability of transitioning over time from certain latent class to another (i.e., from T1 to T2, or from T2 to T3). Following the guidelines from Morin and Litalien (2017), the transitions were estimated using the manual auxiliary 3-step approach (Asparouhov and Muthén, 2014). The benefit of this approach is that the latent classes remain unchanged when combined when distal outcomes are added to the model. It is noteworthy, that as our PRF measurement methods varied at each time point (T1 vs. T2 and T3), we did not test for configural (the number of profiles) or structural (the similarity of the profiles) assumptions in the LPA.

Regarding our research question about the associations between maternal PRF and child development, we tested, first, the effects of the cross-sectional latent classes (from LPA) on child’s internalizing and externalizing problems, socio-emotional competence, and effortful control. Second, we tested the effects of the PRF trajectories (from LTA) on the same outcomes. The PRF trajectories were defined as the group of mothers that had the same combination of the latent classes over time (T1-T2-T3). For both statistical and practical reasons, we focused only on the trajectories that would have sufficient group sizes (after considering attrition) to achieve statistical power (> 0.80) in analyzing the child outcomes (i.e., *n* > 54 when assuming emergence of 6 distinct trajectories, α = 0.05 and medium effect size *f* = 0.25). The 3-step approach was used to conduct test the effects of the cross-sectional PRF profiles and trajectories on the child outcome (Asparouhov and Muthén, 2014; Morin and Litalien, 2017). Wald test (*w*) was used to perform the pairwise tests between the latent classes. Due to high number of comparisons, the *p*-values were corrected using the graphically sharpened False Discovery Rate method (Benjamini and Hochberg, 2000). The background variables were used as covariates in these analyses.

Finally, regarding our research question about the statistical predictors of the maternal PRF, first, we tested the background variables (i.e., child’s gender, parity, maternal education and maternal age) and infant temperament (i.e., surgency, self-regulation/orienting, negative affectivity) as statistical predictors of the PRF profiles. This was done using the 3-step approach in Mplus. Second, we tested the background variables and infant temperament as statistical predictors of the PRF trajectories. Due to high complexity of the modeling and technical estimation issues, that was conducted in SPSS using the nominal classification saved from Mplus.

Attrition analysis showed that missingness in the data did not occur completely at random, Little’s test χ^2^(2813) = 3571.28, *p* < 0.001. Further tests suggested that missingness at T3 associated with mothers younger age, *p* < 0.001, primiparity, *p* < 0.001, and lower education level, *p* < 0.001. It also associated with mother’s higher Certainty, higher Interest, and lower Dynamics at T1, all *p*’s < 0.030, and higher Certainty at T2, *p* = 0.010. Finally, it associated with infant’s higher surgency at T2, *p* = 0.001, but not with negative affectivity or regulation/orienting, *p*’s > 0.116. It is important to notice the attrition here as limitation to our study.

## Results

### Descriptive statistics

Means, standard deviations and correlations between the study variables are shown in Table 1. Regarding the maternal PRF variables, there was moderate continuity from pregnancy (T1) Certainty to Certainty during infancy at 6 months (T2) (*r* = 0.46), and from Interest at T1 and Dynamics at T1 to Interest at T2 (*r*’s > 0.33). Moreover, there was moderate continuity from T2 to child’s age of 2 years (T3) within both Certainty (*r* = 0.64) and Interest (*r* = 0.65). To further describe the relations between the study variables, we regressed the background variables and infant temperament at T2 on the child outcomes at T3 (for details, see Supplementary Material S3). In short, children of older mothers showed both lower competence and effortful control. Mother’s higher education associated with children’s lower externalizing and internalizing problems, and higher competence and effortful control. Multiparity associated with children’s higher effortful control. Girls, compared to boys, were reported to display lower externalizing and internalizing problems, and higher competence and effortful control. Regarding infant temperament, higher surgency associated with lower internalizing problems, and higher competence and effortful control. Negative affectivity associated with higher externalizing and internalizing problems, and lower competence and effortful control. Finally, higher regulation/orienting associated with lower externalizing problems, and higher competence and effortful control.

**Table 1:** Descriptive information and correlations between the study variables.

			Correlations			*M*	*SD*	1		2		3		4		5		6		7		8		9		10		11		12		13		14		15		16		17		18
1. Maternal age	30.24	4.71	1.00																																		
2. Maternal education	1.95	0.84	0.31	***	1.00																																
3. Parity status^a^	0.49	0.50	0.30	***	0.02		1.00																														
4. Child’s gender^b^	0.48	0.50	−0.01		0.00		−0.01		1.00																												
5. Certainty T1	2.72	0.93	−0.09	***	−0.21	***	0.05	*	0.03		1.00																										
6. Reflection T1	4.44	1.10	−0.17	***	−0.05	*	−0.30	***	−0.01		0.15	***	1.00																								
7. Dynamics T1	4.20	1.10	−0.19	***	−0.03		−0.22	***	−0.01		0.12	***	0.62	***	1.00																						
8. Certaintly T2	3.67	1.10	−0.03		−0.11	***	0.04		0.00		0.47	***	0.09	***	0.01		1.00																				
9. Interest T2	5.91	0.88	0.00		0.11	***	−0.16	***	0.02		0.00		0.42	***	0.33	***	0.14	***	1.00																		
10. Certainty T3	3.65	1.07	0.01		−0.07	**	0.01		0.04		0.44	***	0.07	**	0.01		0.64	***	0.09	***	1.00																
11. Interest T3	5.97	0.80	−0.06	*	0.14	***	−0.14	***	−0.05	*	−0.07	**	0.35	***	0.29	***	0.06	*	0.63	***	0.09	***	1.00														
12. Externalizing T3	2.61	1.98	−0.05		−0.05		−0.02		−0.16	***	−0.05	*	0.00		0.05	*	−0.05	*	−0.07	*	−0.13	***	−0.05		1.00												
13. Internalizing T3	3.40	2.48	−0.03		−0.03		0.03		0.03		−0.03		0.03		0.07	**	−0.10	***	−0.04		−0.14	***	−0.02		0.31	***	1.00										
14. Competence T3	18.06	2.46	0.02		0.00		0.08	**	0.06	*	0.11	***	0.08	**	0.02		0.20	***	0.19	***	0.25	***	0.19	***	−0.36	***	−0.22	***	1.00								
15. Effortful control T3	4.96	0.56	−0.04		0.06	*	0.01		0.17	***	0.05		0.12	***	0.08	**	0.09	***	0.21	***	0.12	***	0.21	***	−0.22	***	−0.13	***	0.52	***	1.00						
16. Surgency T2	4.75	0.71	−0.04		−0.12	***	−0.04		−0.05	*	0.07	**	0.18	***	0.10	***	0.14	***	0.17	***	0.10	***	0.14	***	0.02		−0.05		0.30	***	0.25	***	1.00				
17. Negative affectivity T2	3.02	0.76	−0.03		0.02		0.02		0.06	**	−0.02		0.08	**	0.14	***	−0.22	***	−0.02		−0.13	***	0.03		0.18	***	0.30	***	−0.17	***	−0.06	*	0.08	**	1.00		
18. Regulation/orienting T2	5.27	0.65	0.04		−0.06	*	−0.02		0.03		0.07	**	0.12	***	0.05	*	0.25	***	0.22	***	0.17	***	0.16	***	−0.11	***	−0.12	***	0.43	***	0.28	***	0.47	***	−0.25	***	1.00

a 0, primiparous; 1, multiparous.

b 0, girl; 1, boy. Child outcome (12–15) and temperament (16–18) variables are based on averaged reports provided by both mothers and fathers. T1, Pregnancy; T2, Child’s Age of 6 Months; T3, Child’s Age of 2 Years.

* *p* < 0.05;

** *p *< 0.01;

*** *p* < 0.001.

### Identification of cross-sectional parental reflective functioning profiles

Regarding the optimal number of the PRF profiles (i.e., latent classes) at T1, T2 and T3, the fit indices and tests provided somewhat mixed results. As shown in Table 2, BIC indicated 5 classes at T1 and 6 classes at T3 but found no optimal number of classes at T2. In contrast, VLMR indicated 4 classes at T1, 3 classes at T2, and 2 classes at T3. Yet, AIC, AICC, ABIC or BLRT did not indicate optimal number of classes when estimating solutions from two to seven classes. After screening the theoretical meaningfulness (e.g., interpretability and distinctiveness) of the latent classes, we decided to choose 4 classes at T1, 3 classes at T2, and 2 classes at T3, as indicated by VLMR. These solutions provided plausible clarity of the classification, as indicated by entropy values at of 0.68, 0.80 and 0.74, for T1, T2 and T3, respectively.

**Table 2:** Fit Indices from the cross-sectional latent class analyses.

	1 class	2 classes	3 classes	4 classes	5 classes	6 classes	7 classes
At pregnancy (T1; *n =* 2,573)							
AIC	21906.22	20974.99	20614.72	20540.77	20477.66	20455.38	20443.12
AICC	21906.25	20975.08	20614.89	20541.04	20478.05	20455.93	20443.85
BIC	21941.34	21033.52	20696.66	20646.12	20606.42	20607.55	20618.71
ABIC	21922.28	21001.75	20652.18	20588.93	20536.52	20524.94	20523.39
VLMR (*p*)		0.000	0.000	0.000	0.080	0.090	0.390
BLRT (*p*)		0.000	0.000	0.000	0.000	0.000	0.000
At the child’s age of 6 months (T2; *n =* 1900)							
AIC	10789.09	10534.97	10421.24	10387.64	10345.85	10306.39	10289.46
AICC	10789.12	10535.03	10421.36	10387.83	10346.14	10306.80	10290.00
BIC	10811.29	10573.82	10476.74	10459.78	10434.65	10411.83	10411.55
ABIC	10798.58	10551.58	10444.97	10418.48	10383.82	10351.47	10341.66
VLMR (*p*)		0.000	0.000	0.180	0.210	0.040	0.000
BLRT (*p*)		0.000	0.000	0.000	0.000	0.000	0.000
At the child’s age of 2 years (T3; *n =* 1,367)							
AIC	7761.08	7624.31	7561.18	7522.83	7501.73	7470.31	7457.35
AICC	7761.11	7624.39	7561.34	7523.10	7502.13	7470.87	7458.10
BIC	7781.96	7660.85	7613.38	7590.69	7585.26	7569.49	7572.20
ABIC	7769.26	7638.62	7581.62	7549.40	7534.43	7509.14	7502.31
VLMR (*p*)		0.000	0.190	0.100	0.010	0.380	0.620
BLRT (*p*)		0.000	0.000	0.000	0.000	0.000	0.000

AIC, Akaike’s Information Criterion; AICC, corrected AIC; BIC, Bayesian Information Criterion; ABIC, adjusted BIC; BLRT, bootstrapped log likelihood ratio test; VLMR, Vuong-Lo–Mendell–Rubin Test.

**Figure 1:**
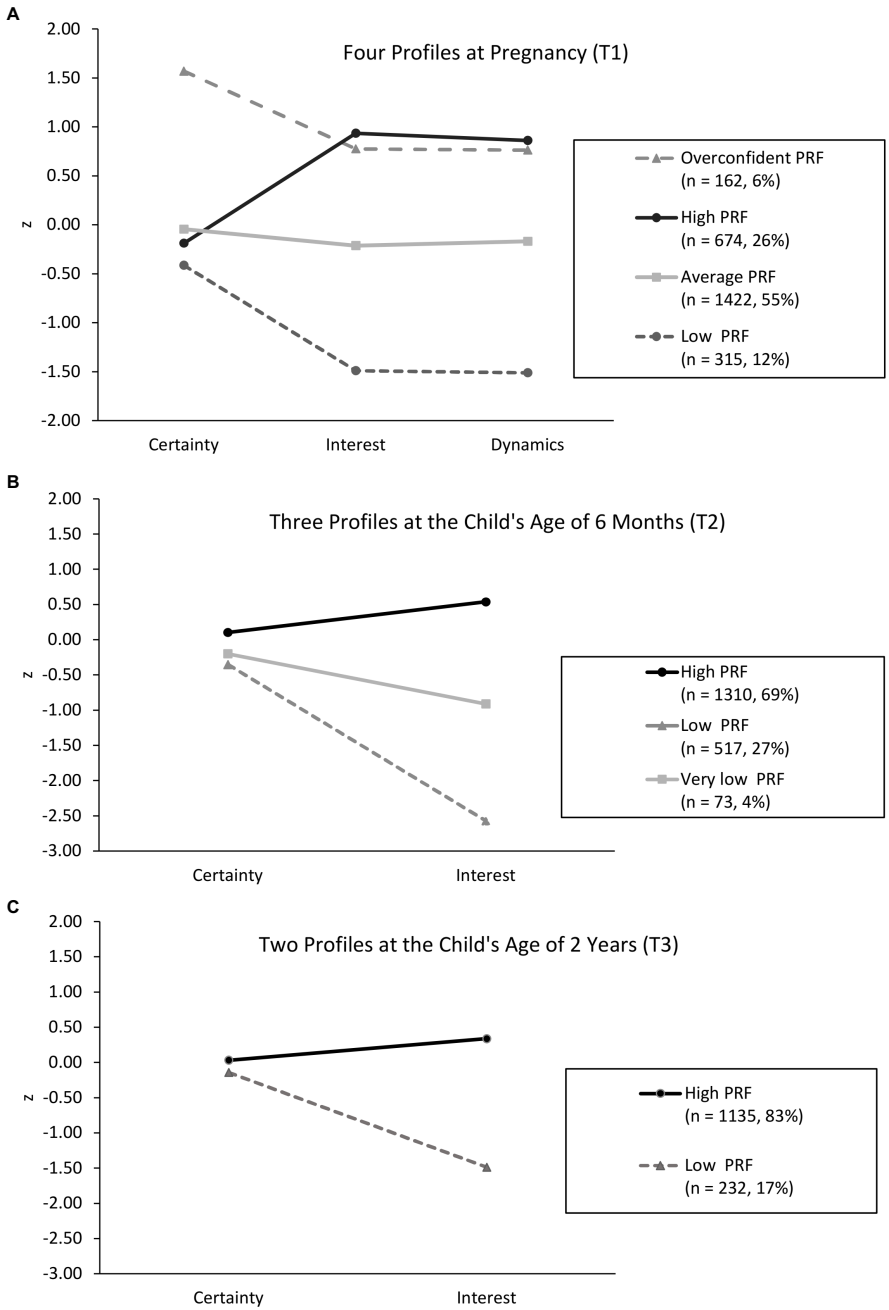
**(A–C)** Cross-sectional maternal PRF profiles.

We interpreted and labeled the latent classes based on the standardized means of the indicator variables. As shown in Figure 1A, during pregnancy (T1), the first profile comprised 55% (*n =* 1,422) of the sample and involved scores close to the mean in Certainty, Interest, and Dynamics. Thus, we labeled it as “Average PRF” profile. The second profile (26%; *n =* 674) involved high scores in Interest and Dynamics, yet Certainty being close to the mean. Thus, we labeled it as “High PRF” profile. The third profile (12%; *n =* 315) involved low scores on Interest and Dynamics, and slightly decreased Certainty. Thus, we labeled it “Low PRF” profile. The fourth profile (6%; *n =* 162) involved exceptionally high score on Certainty, and high score also in Interest and Dynamics. Thus, we labeled it as “Overconfident PRF” profile.

As shown in Figure 1B, at the child’s age of 6 months (T2), the first profile (69%; *n =* 1,310) involved high scores on Certainty and Interest. Thus, we labeled it as “High PRF” profile. The second profile (27%; *n =* 517) involved low Interest. Thus, we labeled it as “Low PRF” profile. The third profile (4%; *n =* 73) involved very low scores on Interest. Thus, we labeled it as “Very low PRF” profile. In all profiles the scores of Certainty were close to mean (within the range of 0.5 SD).

Finally, as shown in Figure 1C, at the child’s age of 2 years (T3), the first profile (83%; *n =* 1,135) involved high scores on Interest. Thus, we labeled it as “High PRF” profile. The second profile (17%; *n =* 232) involved low Interest. Thus, we labeled it as “Low PRF” profile. In both profiles, again, the scores of Certainty were close to average.

### Predictors of the cross-sectional parental reflective functioning profiles

To examine how the background variables and infant temperament statistically predicted the cross-sectional latent class membership, we used the logistic regression in the 3-step analysis. To simplify the analyses and interpretation, we used the “High PRF” profile as a reference group at T1, T2 and T3. As shown in Table 3, higher maternal age associated with the “Average PRF,” “Overconfident PRF,” and “Low PRF” profiles at T1. Mother’s low education level associated with the “Average PRF” and “Overconfident PRF” profiles at T1, as well as with the “Low PRF” profile at both T2 and T3. Being multiparous associated negatively the “High PRF” profile at T1, T2 and T3. Child being a boy (versus girl) associated with the “Low PRF” profile both at T2 and T3. Regarding infant temperament, both low surgency and low negative affectivity associated with the “Average PRF” and “Low PRF” profiles at T1. Furthermore, infant’s low regulation/orienting associated with the “Low PRF” at T1, T2 and T3, as well as with the “Very low PRF” at T2.

**Table 3:** Predictors of membership in the cross-sectional latent PRF profiles.

	At pregnancy (T1)									At the child’s age of 6 months (T2)			At the child’s age of 2 years (T3)		
Average PRF			Overconfident PRF			Low PRF			Low PRF			Very low PRF			Low PRF		
	B	SE	p	B	SE	p	B	SE	p	B	SE	p	B	SE	p	B	SE	p
Maternal age	0.09	0.03	0.001	0.11	0.06	0.045	0.14	0.03	<0.001	−0.02	0.02	0.342	0.03	0.04	0.380	0.05	0.03	0.081
Education level	−0.39	0.13	0.003	−1.31	0.30	<0.001	−0.30	0.16	0.063	−0.32	0.09	0.001	−0.32	0.18	0.076	−0.52	0.13	<0.001
Parity status	1.50	0.25	0.000	1.63	0.44	<0.001	2.35	0.29	<0.001	0.49	0.15	0.002	1.47	0.38	<0.001	0.64	0.21	0.003
Child’s gender	−0.14	0.19	0.482	−0.12	0.39	0.762	0.21	0.24	0.373	0.32	0.15	0.032	−0.47	0.33	0.149	0.56	0.22	0.009
IBQ surgency	−0.67	0.18	<0.001	−0.25	0.35	0.474	−0.65	0.22	0.003	−0.14	0.13	0.274	−0.43	0.27	0.112	0.08	0.19	0.686
IBQ negative affectivity	−0.35	0.14	0.015	−0.26	0.30	0.378	−0.81	0.19	<0.001	−0.04	0.11	0.703	−0.19	0.21	0.367	−0.26	0.16	0.094
IBQ regulation/orienting	−0.08	0.19	0.677	0.41	0.37	0.273	−0.51	0.24	0.030	−0.95	0.15	<0.001	−0.84	0.32	0.008	−0.94	0.23	<0.001

At each time point (T1, T2, and T3) “High PRF” profile is used as the reference group. Values for parity status are 0, primiparous; 1, non-primiparous; Values for child’s gender are 0, girl; 1, boy.

### Cross-sectional parental reflective functioning profiles and child outcomes

To answer our research question regarding the associations between the cross-sectional maternal PRF profiles (T1, T2 or T3) and child outcomes at the age of 2 years (T3), we used the 3-step analyses. In separate runs, the profile membership was independent variable, the child outcomes were the dependent variables, and the background variables and infant temperament were the covariates. To simplify the pairwise comparisons, we used the “High PRF” profile as a reference group in *post hoc* tests. The results showed that the maternal PRF profiles at T1, *w(*3) = 22.165, *p* < 0.001, at T2, *w(*2) = 16.41, *p* < 0.001, and at T3, *w(*3) = 9.80, *p* = 0.002, associated with children’s socio-emotional competence at T3. At T1, children of mothers with the “High PRF” profile showed higher competence compared to the “Average PRF,” *diff =* 0.57, *SE* = 0.23, *p* = 0.014, and “Low PRF,” *diff =* 0.81, *SE* = 0.28, *p* = 0.004, profiles. No difference emerged between the “High PRF” and “Overconfident PRF” profiles, *p* = 0.200. At T2, children of the mothers with the “High PRF” profile showed higher competence compared to the “Low PRF” profile, *diff =* 0.69, *SE* = 0.18, *p* < 0.001. Yet, no difference emerged between the “High PRF” and the “Very low PRF” profiles, *p* = 0.123. Finally, at T3, children of the mothers with the “High PRF” profile were rated to show higher competence compared to the “Low PRF” profile, *diff =* −0.73, *SE* = 0.24, *p* = 0.002. Against our expectations, the PRF profiles at T1, *p’s* > 0.234, at T2, *p’s* > 0.076, or at T3, *p’s* > 0.077, did not associate with children’s internalizing or externalizing problems, nor with effortful control.

### Transitions over time and the parental reflective functioning trajectories

**Figure 2:**
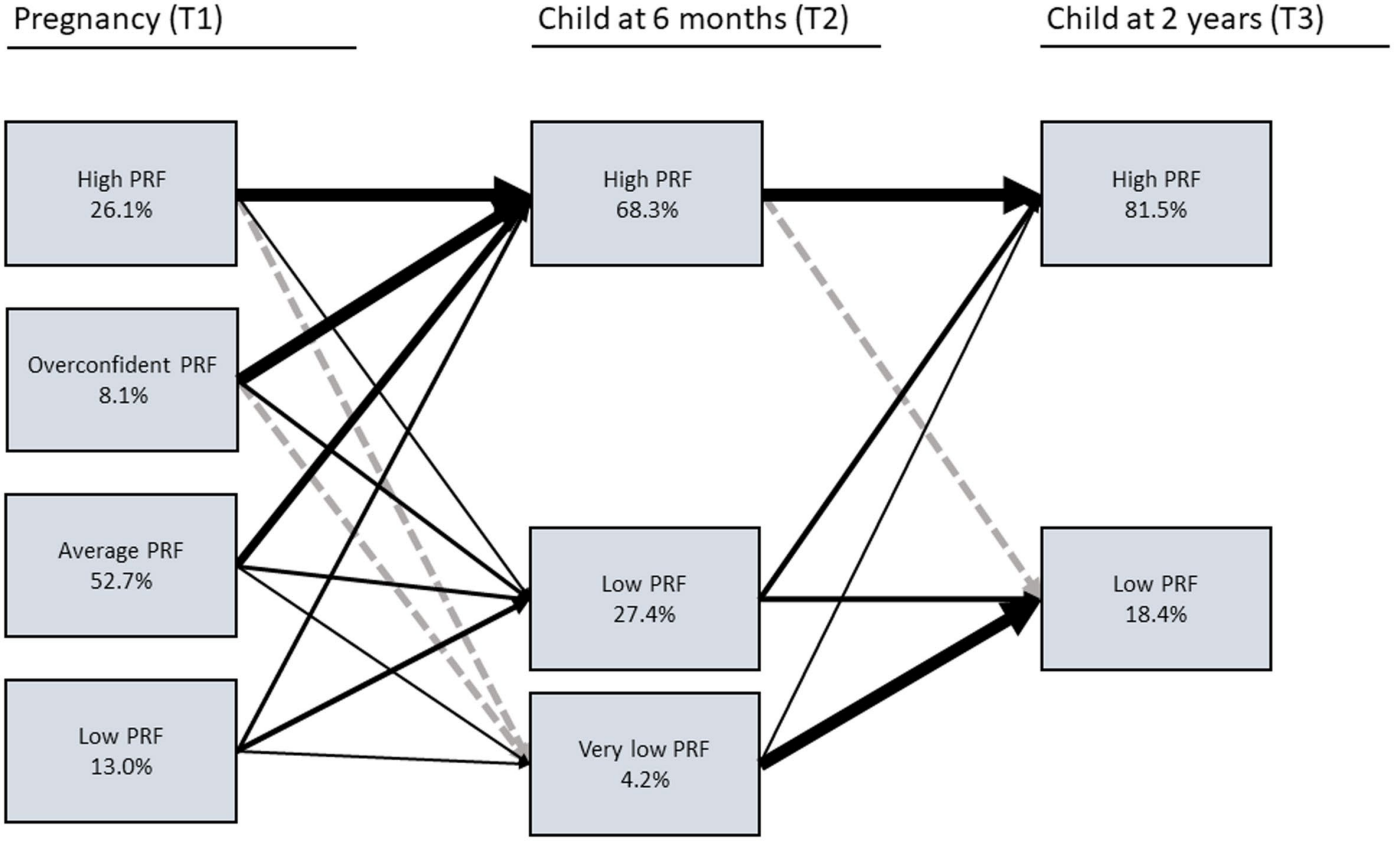
Transitions between the maternal PRF profiles. The width of the arrows depict the probability of the transitions (larger arrows indicate higher transitional probability). Broken arrows indicate no transitions between the profiles.

To answer our research questions regarding mother’s pathways of PRF, we combined the cross-sectional PRF classes in Latent Transition Analysis. The longitudinal transitional probabilities are depicted in Table 4 and Figure 2. In summary, there was substantial continuity in the maternal PRF profiles: Most mothers who had “High PRF” profile at one time point had very high probability of belonging to the same class at the subsequent timepoint, p(T1|T2) = 0.96 and p(T2|T3) = 1.00. In other words, it was extremely unlikely to transition from “High PRF” to a profile that represented lower levels of PRF. There was also continuity in “Low PRF” profile, p(T1|T2) = 0.52 and p(T2|T3) = 0.86. Yet, substantial group of mothers with “Low PRF” also transitioned to “High PRF,” p(T1|T2) = 0.29 and p(T2|T3) = 0.54. Relatedly, mothers with “Very low PRF” at T2 most likely had “Low PRF” at T1, p(T1|T2) = 0.19, and majority of mothers with “Very low PRF” at T2 transitioned to “Low PRF” at T3, p(T2|T3) = 0.86. Over half of the mothers with “Average PRF” at T1 transitioned to “High PRF” at T2, p(T1|T2) = 0.63, yet third of these mothers transitioned to “Low PRF” at T2, (T1|T2) = 0.34. Finally, most mothers with “Overconfident PRF” at T1 transitioned to “High PRF” at T2, p(T1|T2) = 0.80, and one fifth of the mothers transitioned to “Low PRF” at T2, (p(T1|T2) = 0.20.

**Table 4:** Estimated transitional probabilities for the maternal PRF profiles.

	Child’s age of 6 months (T2)			Child’s age of 2 years (T3)		
	High PRF	Low PRF	Very low PRF		High PRF	Low PRF
Pregnancy (T1)				Child’s age of 6 months (T2)	
Overconfident PRF	0.80	0.20	0.00	High PRF	1.00	0.00
High PRF	0.96	0.04	0.00	Low PRF	0.46	0.54
Average PRF	0.63	0.34	0.03	Very low PRF	0.14	0.86
Low PRF	0.29	0.52	0.19			

The estimated probability of belonging to high, overconfident, average and low profiles at T1 were 0.08, 0.26, 0.53, and 0.13, respectively.

To depict the most common transitional pathways, that is the maternal PRF trajectories, we tabulated the frequencies of the different latent class combinations (see Supplementary Material S4). We choose the six largest trajectories, accounting 84% of the cases, to be examined in more detailed analyses. We labeled the maternal PRF trajectories as Average_T1_-High_T2_-High_T3_ (AHH; 41%, *n =* 1,100), High_T1_-High_T2_-High_T3_ (HHH; 22%, *n =* 598), Average_T1_-Low_T2_-High_T3_ (ALH; 9%, *n =* 249), Low_T1_-High_T2_-High_T3_ (LHH; 6%, *n =* 170), Overconfident_T1_-High_T2_-High_T3_ (OHH; 5%, *n =* 137), and Average_T1_-Low_T2_-Low_T3_ (ALL; 3%, *n =* 80). For these trajectories, complete data was available from *n =* 49 to *n =* 418 cases, yet, the latent analyses regarding child outcomes utilized the whole dataset (*n =* 2,536). Finally, we combined all the other smaller (*n* ≤ 66) trajectories into one mixed group of trajectories, labeled as “Mixed trajectories” (MIX; 16%, *n =* 223).

### Predictors of the parental reflective functioning trajectories

To examine how the background variables and infant temperament statistically predicted belonging to the maternal PRF trajectories (T1-T3), we ran a multinomial regression analysis in SPSS. The results showed that maternal age, χ^2^(6) = 12.66, *p* = 0.049, education level, χ^2^(6) = 33.39, *p* < 0.001, and parity status, χ^2^(6) = 74.02, *p* < 0.001, as well as infant negative affectivity, χ^2^(6) = 21.61, *p* < 0.001, and regulation/orienting, χ^2^(6) = 56.48, *p* < 0.001, associate with belonging to the trajectories. Table 5 presents the mean estimates of the statistical predictors within each trajectory group and detailed pairwise tests (with FDR corrections for 21 *p*-values).

**Table 5:** Predictors (with mean estimates) of membership in the maternal PRF trajectories.

	Low_T1_-high_T2_-high_T3_ (LHH)		Average_T1_-high_T2_-high_T3_ (AHH)		Average_T1_-low_T2_-low_T3_ (ALL)		Average_T1_-low_T2_-high_T3_ (ALH)		Overconfident_T1_-high_T2_-high_T3_ (OHH)		High_T1_-high_T2_-high_T3_ (HHH)		Mixed trajectories (MIX)	
	*M*	*SE*	*M*	*SE*	*M*	*SE*	*M*	*SE*	*M*	*SE*	*M*	*SE*	*M*	*SE*
Maternal age	31.78	0.32	30.89	0.13	30.94	0.47	30.04	0.28	29.95	0.49	29.51	0.18	31.11	0.26
Education level	1.99^c^	0.06	2.05^c^	0.03	1.88^ab^	0.10	1.87^ab^	0.05	1.70^b^	0.07	2.02^c^	0.03	2.06^ac^	0.05
Parity status	0.70^ab^	0.04	0.51^b^	0.02	0.54^ab^	0.06	0.50^ab^	0.03	0.46^b^	0.04	0.27^c^	0.02	0.59^a^	0.03
Child’s gender	0.49	0.04	0.47	0.01	3.00	0.05	0.49	0.03	0.44	0.04	0.46	0.02	0.50	0.03
IBQ surgency	4.68	0.08	4.71	0.02	4.57	0.07	4.63	0.04	4.88	0.07	4.85	0.03	4.60	0.04
IBQ negative affectivity	2.82^a^	0.08	2.97^cd^	0.03	3.00^abcd^	0.07	3.15^bc^	0.04	3.03^bcd^	0.08	3.10^b^	0.03	3.00^a^	0.04
IBQ regulation/orienting	5.25^ac^	0.06	5.26^cd^	0.02	4.99^ab^	0.06	5.05^ab^	0.04	5.41^d^	0.06	5.28^cd^	0.03	5.05^b^	0.03

Mean values on rows that have no superscript in common indicate statistically significant differences based on the results from multinomial regression analysis (*p* < 0.05). Values for parity status are 0, primiparous; 1, non-primiparity; for child’s gender 0, girl; 1, boy; 1, low education; 2, middle education; 3, high education.

In summary of Table 5, high education level associated positively with the LHH, AHH and HHH trajectories, and negatively with the OHH trajectory. The associations between education level and the ALL, ALH and MIX fell between these groups. Primiparity was strongly and positively associated with the HHH compared to the other trajectories, yet primiparity associated positively also with the AHH and OHH trajectories. In contrast, multiparity associated positively with the MIX trajectories. The associations between parity and LHH, ALL and ALH fell between these groups. Regarding infant temperament, negative affectivity associated positively with the HHH trajectory. In contrast, negative affectivity associated negatively with the LHH and MIX trajectories. The associations between negative affectivity and AHH, ALH and OHH fell between these groups. Finally, child’s high regulation/orienting associated positively with the AHH, OHH, and HHH trajectories. In contrast, regulation/orienting associated negatively with the ALL, ALH and MIX trajectories. The associations between regulation/orienting and LHH fell between these groups. While mothers in the HHH trajectory were youngest, the differences were non-significant after FDR correction. Altogether, Cox and Snell Pseudo R^2^ indicated that these factors accounted 14% of belonging to the PRF trajectories. Child’s gender, χ^2^(6) = 5.95, *p* = 0.429, and surgency, χ^2^(6) = 12.14, *p* = 0.059, did not associate with the trajectories.

### Parental reflective functioning trajectories and child outcomes

**Figure 3:**
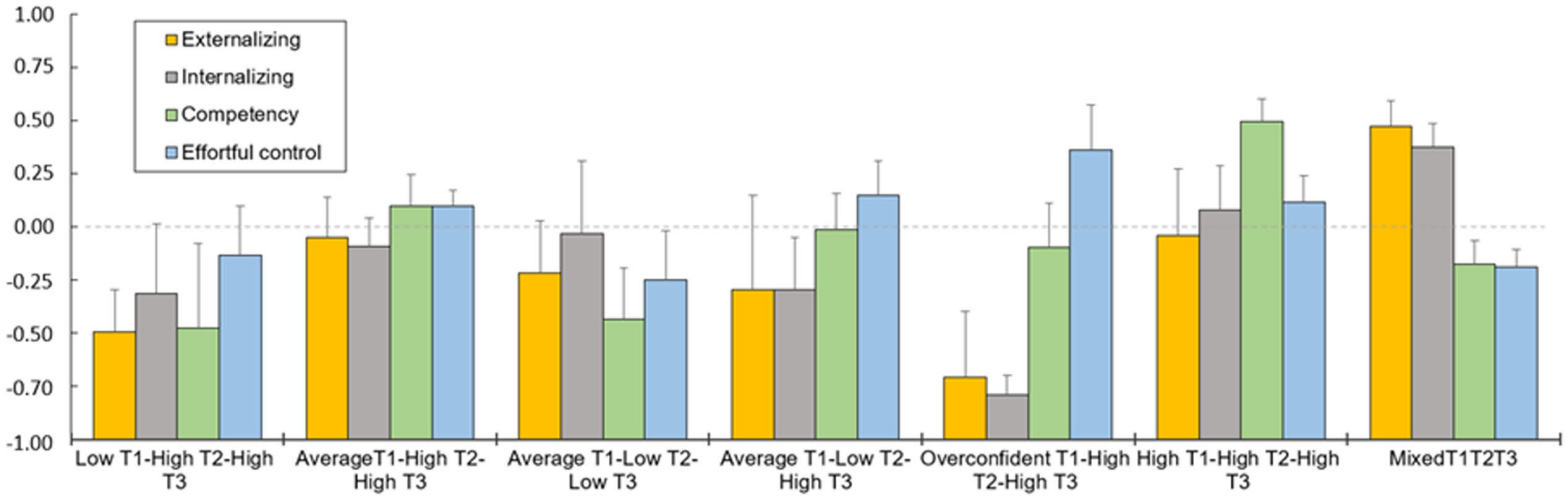
Associations between the maternal PRF trajectories and child outcomes. T1, pregnancy; T2, Child’s age of 6 months; T3, Child’s age of 2 years.

To answer our final and main research questions regarding the associations between the maternal PRF trajectories (T1-T3) and child outcomes at the age of 2 years (T3) we used the 3-step analysis for the trajectories. Omnibus tests showed that the seven PRF trajectories associated with children’s externalizing problems, *W(*6) = 38.49, *p* < 0.001, internalizing problems *w(*6) = 90.39, *p* < 0.001, effortful control, *W(*6) = 13.95, *p* = 0.030, and socio-emotional competence, *W(*6) = 32.55, *p* < 0.001. The mean estimates of the child outcomes are shown in Figure 3, as well as in Table 6 with the results of pairwise tests (with FDR corrections for 84 *p*-values). Pairwise tests showed, first, that children from the MIX trajectories had higher externalizing problems compared to children from the LHH, AHH, ALL and OHH trajectories. Second, children from the MIX trajectories had higher internalizing problems compared to children from the AHH and OHH trajectories. Moreover, children from the OHH trajectory had lower internalizing problems compared to the AHH and HHH. Third, children from the HHH trajectory had higher socio-emotional competence compared to children from the ALL, ALH and MIX trajectories. Finally, while uncorrected *p*-values indicated together with the omnibus test that children from MIX trajectories had lower effortful control compared to AHH and OHH, these differences were not significant after the FDR correction, *p’s* > 0.060. The background variables and infant temperament were used as covariates in this analysis.

**Table 6:** Associations between the maternal PRF Trajectories (T1-T3) and child outcomes at the age of 2 years (T3).

	Low_T1_-high_T2_-high_T3_ (LHH)		Average_T1_-high_T2_-high_T3_ (AHH)		Average_T1_-low_T2_-low_T3_ (ALL)		Average_T1_-low_T2_-high_T3_ (ALH)		Overconfident_T1_-high_T2_-high_T3_ (OHH)		High_T1_-high_T2_-high_T3_ (HHH)		Mixed trajectories (MIX)	
	*M*	*SE*	*M*	*SE*	*M*	*SE*	*M*	*SE*	*M*	*SE*	*M*	*SE*	*M*	*SE*
Externalizing	0.50^a^	0.20	0.95^a^	0.19	0.78^a^	0.25	0.71^ab^	0.44	0.29^a^	0.31	0.96^ab^	0.31	1.47^b^	0.12
Internalizing	0.69^abc^	0.33	0.91^b^	0.13	0.97^abc^	0.34	0.71^abc^	0.24	0.21^a^	0.10	1.08^bc^	0.21	1.38^c^	0.11
Competence	0.52^ab^	0.40	1.10^ab^	0.15	0.57^b^	0.24	0.99^b^	0.17	0.91^ab^	0.21	1.50^a^	0.11	0.82^b^	0.11
Effortful control	0.87	0.23	1.10	0.08	0.75	0.23	1.15	0.16	1.36	0.21	1.12	0.13	0.81	0.08

Estimates of child outcomes are controlled for the demographic and child related variables. Mean values on rows that have no superscript in common are significantly different from each other (p < 0.05). Based on model fit, unequal residual variances and covariances were allowed between the trajectory groups. T1, pregnancy; T2, Child’s age of 6 months; T3, Child’s age of 2 years.

## Discussion

Our aims in the current study were, first, to chart the pathways in maternal PRF from pregnancy to child’s age of 2 years, second, to study their associations with child development, and third, to examine their associations with contextual demographic and child related factors. The PRF profiles largely reflected variance in the Interest dimension, that is, mothers’ curiosity towards the child’s experiences, perspective and mental states. During pregnancy, Interest closely covaried with Dynamics, indicating that the mothers who were curious on their child’s mind also reflected on their own past, present and future experiences. As hypothesized, there was a general trend of progression towards higher levels of PRF over time, yet, the results also evidenced high variability in the PRF trajectories. Both the PRF profiles and trajectories associated with children’s social–emotional competence in toddlerhood, and furthermore, the trajectories associated with children’s internalizing and externalizing problems. As hypothesized, children from the stable high PRF trajectory (i.e., High_T1_-High_T2_-High_T3_) had highest levels of social–emotional competence, involving for example, prosocial peer relations, empathy and play skills. Furthermore, children from the more atypical PRF trajectories (i.e., mixed) showed relatively high internalizing and externalizing problems. Against our hypothesis, the PRF trajectories did not associate with children’s effortful control in toddlerhood. Finally, the PRF trajectories had various associations with background variables (e.g., parity) and infant temperament. Our novel results illuminate the naturally occurring maternal PRF pathways, highlight and delineate its potential significance on child development, and raise important questions about the nature of self-reported PRF.

### Maternal parental reflective functioning profiles

Mentalization literature has described the multitude of ways how people may relate to own and others mental states (Fonagy et al., 2002, 2012; Fonagy and Luyten, 2009). In the current study, we used latent analyses to empirically identify distinct PRF profiles among mothers. The analyses identified four profiles during pregnancy (i.e., Overconfident, High, Average and Low PRF), three at the child’s age of 6 months (i.e., High, Low and Very Low PRF), and two at the child’s age of 2 years (i.e., High and Low PRF). While we had no hypotheses about the content of the PRF profiles, it was somewhat surprising that most of the profiles had equal values on Certainty. In other words, mothers in all profiles were relatively certain about knowing their child’s mental states. In contrast, the profiles differed largely in Interest, that is, in curiosity towards the child’s mind. These patterns of results indicate that the mothers with the high PRF profile were confident and keenly curious about their child’s mind. In contrast, mothers with the lower PRF profiles (e.g., Very Low PRF and Low PRF) were also confident knowing their child’s mind but had less interest and curiosity on it. This suggests that the mothers with the lower PRF profiles may have lacked awareness about the opaqueness and uncertainty in knowing their child’s mental states.

The Overconfident PRF profile (6%) was an intriguing exception from the other profiles by showing very high score on Certainty. This was accompanied by high scores on Interest and Dynamics. High scores on Certainty have been suggested to indicate hypermentalizing, that is, a tendency to overattribute mental states to others (Slade, 2002, Slade et al., 2010). Yet, previous variable-oriented PRFQ studies have provided ambiguous evidence regarding its role, some linking high scores with maladaptive (e.g., intrusiveness; Rostad and Whitaker, 2016) and some with adaptive parenting (e.g., high self-efficacy; De Roo et al., 2019). In our person-oriented analyses, the Overconfident profile emerged only during pregnancy, but not after the child’s birth. Thus, it may be part of idealized prenatal expectations of parenthood that typically become more realistic in the postnatal period (Flykt et al., 2014).

PRF during pregnancy is a complex and challenging task because there are not yet actual interactions with the child to build upon (Slade et al., 2009; Slade, 2011). The prenatal PRF process emphasizes the role of mother’s already existing mental representations about being a young child and becoming parenthood, involving own experiences in past and present relationships (Pajulo et al., 2015). In line with this, the Interest and Dynamics dimensions co-occurred at the same level in the four prenatal profiles, indicating that mothers who were oriented towards the experiences of the fetus-baby also reflected on their own life experiences and relationships. In general, this concurs with research showing that mother’s recollections about receiving care from own parents associate with higher maternal-fetal attachment during pregnancy (Sacchi et al., 2021). Further, the postnatal PRF has been found to mediate between prenatal and postnatal mother-to-child attachment (Pazzagli et al., 2022). As such, the prenatal PRF seems to be an integral part of forming the early bond with the child and likely strengthens both the postnatal PRF and the mother-to-child attachment.

### Longitudinal transitions and parental reflective functioning trajectories

The overall progression of maternal PRF was positive, as during pregnancy only 26% of the mothers had high PRF profile, and the number of mothers with this profile increased to 69% in infancy and to 83% in toddlerhood. This confirms the results of the one previous longitudinal study utilizing PRFQ, suggesting that on average there is a steep increase in PRF capacity after childbirth compared with the prenatal level (Salo et al., 2021). Both the actual interactions with the child and the maturation of child’s expressive skills likely foster the development of parental PRF (Slade et al., 2010; Salo et al., 2021). Importantly, our person-oriented study specified that maternal PRF develops like a skill that, once acquired in relation to the child, is very rarely lost. This was indicated by that of the mothers who had achieved high PRF, one fourth already during pregnancy, almost none transitioned to the profiles representing lower levels of PRF.

During pregnancy the majority (55%) of the mothers belonged to the Average PRF profile. Half of these mothers transitioned to High PRF and one third to Low PRF profiles during the child’s infancy. As such, average PRF during pregnancy does not seem to represent neither a particular risk nor a resiliency factor for later PRF. In contrast, one tenth (12%) of the mothers belonged to Low PRF profile during pregnancy. The profile was characterized by disinterest on fetus-baby’s experiences and lacking reflection of own past, present and future experiences. Half of these mothers remained in the Low PRF profile also during child’s infancy and one fifth transitioned to the Very low PRF profile. Therefore, low prenatal PRF seems to represent a considerable risk for postnatal PRF. Such development seems to easily accumulate, as half of the mothers with low PRF during child’s infancy continued to have low PRF also at the child’s age of 2 years. Finally, it is interesting to note that majority (80%) of the mothers with Overconfident PRF during pregnancy transitioned to High PRF during the child’s infancy. This suggest that rather being a risk, the prenatal overconfidence may have bolstered positive experience of parenthood and high PRF.

To depict and summarize the maternal PRF pathways we combined the most common PRF profiles to six longitudinal trajectories. These accounted 87% of the mothers. The most common trajectories were Average_T1_-High_T2_-High_T3_ (AHH; 41%) and High_T1_-High_T2_-High_T3_ (HHH; 22%), depicting stable high maternal PRF from pregnancy to child’s toddlerhood. Relatively large variance in the prenatal PRF was demonstrated by the Low_T1_-High_T2_-High_T3_ (LHH; 6%) and Overconfident_T1_-High_T2_-High_T3_ (OHH; 5%) trajectories. Despite having low or overconfident PRF during pregnancy, mothers with these trajectories achieved high PRF by the child’s infancy, perhaps aided by the actual interaction experiences with the infant. Finally, the Average_T1_-Low_T2_-Low_T3_ (ALL; 9%) and Average_T1_-Low_T2_-High_T3_ (ALH; 3%) trajectories involved low PRF at some timepoint. Mothers with the ALH trajectory had low PRF at the child’s infancy but obtained high PRF by the child’s toddlerhood, whereas the mothers with the ALL trajectory did not obtain high PRF. Likely, it was common for these mothers to experience some difficulties in their early parenthood, yet the mothers with the ALL trajectory could better overcome these challenges. The smaller trajectories were assigned to a Mixed trajectory (MIX; 13%) group.

### Parental reflective functioning trajectories and child outcomes

In line with our hypothesis, the HHH trajectory associated with child’s high social–emotional competence at the age of 2 years. This result was evident over and above the demographic and child related factors, and it was replicated when analyzing the cross-sectional PRF profiles. Altogether, this hypothesized result aligns with the developmental mentalization model (Sharp and Fonagy, 2008) and with previous empirical research (Camoirano, 2017; Colonnesi et al., 2019; Aldrich et al., 2021), suggesting that high parental PRF can promote child’s social–emotional development, involving child’s own mentalizing skills, interpersonal trust, and empathy.

It is noteworthy, however, that children from the HHH trajectory did not differ from all the other trajectories in terms of their social–emotional competence. The difference emerged in contrast with the ALL, ALH and the MIX trajectories, but not in contrast with the LHH, AHH and OHH trajectories. This pattern of results suggests that low maternal PRF *during pregnancy* does not antecede child’s low social–emotional competence, insofar as the mother obtains high PRF during child’s infancy. However, low PRF *during infancy* seem to antecede child’s low social–emotional competence later, even if the mother obtains high PRF by the child’s toddlerhood. These findings align with the view that the developmental effects of maternal PRF are transmitted within the parent–child interactions during infancy, a period during which child development is highly responsive to the quality of parental caregiving (Zeegers et al., 2017; Szepsenwol and Simpson, 2019).

Some caution is needed, however, concerning inferences about the specific mechanisms underlying the observed associations. Previous research suggests that the effects of maternal PRF on child development occur within the parent–child interactions, involving sensitive co-regulation of infant’s experiences, verbalizing mental states, and scaffolding of child’s behaviors (Sharp and Fonagy, 2008; Camoirano, 2017; Zeegers et al., 2017; Szepsenwol and Simpson, 2019). Inevitably, however, in observational studies also genetic factors may have a role. For example, a twin study showed that approximately 40% of variance in toddlers social–emotional competency in BITSEA is attributable to heritable factors, and 50% to shared environmental influences (Rea-Sandin et al., 2019). Thus, the observed association between mother’s high PRF and child’s social–emotional competencies may be, to some extent, accounted by shared genetic factors between the mother and the child. Relatedly, against our hypothesis, we found no associations between the PRF trajectories and child’s effortful control. Heritability of effortful control is approximately 60%, with little evidence about the influence of shared environment (Willems et al., 2019). This, together with controlling the corresponding temperament dimensions during infancy (i.e., regulation/orienting), may explain why we did not observe any associations between maternal PRF and child’s effortful control.

Regarding externalizing and internalizing problems, children from the MIX trajectories had highest scores on both problem dimensions. Specific conclusions are difficult to make because of the heterogenous nature of the MIX group. All the mothers with very low PRF at child’s infancy, and the trajectories with the most chronically low PRF were included together in the MIX group. Thus, it seems that the more atypical and deviant PRF pathways have strongest association with children’s problem behaviors. Moreover, as differences in children’s problems did not emerge between the six trajectories, the most common trajectories may represent relatively normative variation in the unfolding of the maternal PRF.

Thus, against our hypotheses, children from the HHH trajectory did not have particularly low amount of internalizing or externalizing problems. More unexpectedly, the amount of problems among the children from the HHH trajectory did not differ from neither the trajectories with the highest (i.e., MIX) or the lowest (i.e., OHH) child problems. The reason for this surprising null finding may, first, relate to the young age of the children, as clear manifestations of psychopathology are still rare at this age. Indeed, according to the developmental mentalization model (Sharp and Fonagy, 2008), the effects of child’s own early PRF development on mental health may emerge only during later development through various cascading effects. Second, it is well possible that parental PRF influences how the parents perceive their child. As high PRF involves high attentiveness and openness to child’s cues, the mothers with high PRF may be susceptible to overreport child’s problem behaviors. Such biases could mask any beneficial effects of high PRF on child’s internalizing and externalizing problems and diminish differences between the HHH and the more problematic trajectory groups.

### Parental reflective functioning trajectories and contextual factors

Finally, we examined how the contextual demographic and child related factors associated with the maternal PRF trajectories. First, converging with Salo et al. (2021), we found that mothers with the HHH, LHH, and AHH trajectories had relatively high education level. As high parental education level provides resources during the transition to parenthood (e.g., due to better economic situation), it can help the mothers to obtain and maintain high PRF after child’s birth. Further, converging with Rutherford et al. (2015), mothers with the OHH trajectory had relatively low education level. The specific mechanisms underlying this association is unclear, but it suggests that mothers with low education level may have overly optimistic and perhaps unanalytical approach to perceive own parenthood. Second, converging with Salo et al. (2021) and Pajulo et al. (2015), we found primiparity to associate with belonging to HHH, AHH and OHH trajectories. The first-time transition to parenthood is often an intense psychosocial and biological process, and this likely bolsters the development of parental PRF. At the same time, multiparous mothers may be occupied (or even burdened) by the caretaking responsibilities of the older children, leaving less mental resources to focus on PRF. Aligning with such a view, multiparity associated with belonging to the MIX trajectory group. Finally, maternal age did not associate with the PRF trajectories, yet older age associated with the lower PRF profiles during pregnancy.

Regarding infant temperament at the age of 6 months, high regulation/orienting associated with belonging to AHH, OHH and HHH trajectories, whereas low regulation/orienting associated with belonging to ALL, ALH and MIX trajectories. Paralleling previous research (Kiff et al., 2011; Wittig and Rodriguez, 2019), infant’s high regulatory capacities (e.g., soothability and attentiveness) can make it easier for the mothers to obtain and maintain high PRF, whereas low regulatory capacities can increase caregiver burden and frustration, and thereby hinder the PRF. Interestingly, infant’s high negative affectivity associated with belonging to the HHH trajectory, and low negative affectivity with belonging to LHH and MIX trajectories. Such direction of associations is surprising, as infant’s high negative affectivity (e.g., fearfulness and slow recovery) has been previously found to evoke negative parenting (Kiff et al., 2011; Wittig and Rodriguez, 2019). Yet, considering the process of PRF, it is likely that highly mentalizing mothers are highly attentive towards their child’s cues of distress and thus susceptible for overreporting. Alternatively, it is also possible that infant’s tendency to express negative emotions motivate the mothers to mentalize the child, for example, to determine the source of infant distress. Infant surgency (e.g., activity and laughter) did not associate with the PRF trajectories.

Altogether, these results on PRF align with previous research on early motherhood (e.g., McNamara et al., 2019) and with the theoretical view that early parenting is influenced by both contextual and child related factors (Sameroff, 2010; Kiff et al., 2011). Yet, we did not consider mother’s other characteristics. It would have been theoretically interesting to consider for example mother’s mental health, attachment, and traumatic childhood experiences. Inclusion of such factors would have helped to understand the psychological context of mothers PRF and to disseminate which maternal characteristics drive the effects on child development. For example, depression is known to associate with both impaired PRF abilities (Bora and Berk, 2016) and insensitive maternal caregiving (Bernard et al., 2018), and at the same time, challenges in early parenting can heighten maternal depression (Thomason et al., 2014). Eventually, maternal depression influences child development through both complex genetic and environmental pathways (Natsuaki et al., 2014). Partitioning such effects was not possible within the scope of the current study due to already high complexity of the models. It will be an important area of future research to test how maternal PRF and other maternal characteristics overlap, influence each other, and together explain variance in child development.

### General considerations

Our person-oriented analyses showed that maternal PRF development is characterized by both stability and changes. The stability was mostly accounted by the large group of mothers who experienced continuously high (or at least average) PRF from pregnancy to child’s toddlerhood. While many mothers advanced from low to high PRF, a minority of mothers remained consistently at the low levels. From practical and clinical perspective, mother’s low PRF during pregnancy may be an important sign of need for preventive and supportive interventions, as it antecedes low postnatal maternal PRF with potential significance on child development.

According to mentalization theory, early experiences within the caregiver-child interactions are important for developing child’s own PRF abilities and social understanding (Fonagy, 2006; Sharp and Fonagy, 2008). In line with this, we found a specific association between consistently high maternal PRF and children’s social–emotional competencies. This conforms also with the larger developmental literature, involving randomized experiments (Jeong et al., 2021), according to which sensitive and high-quality caregiving relationships are beneficial for children’s social and emotional development (Camoirano, 2017; Colonnesi et al., 2019; Aldrich et al., 2021). Surprisingly, and departing from the results of previous research, we did not find a beneficial effect of high maternal PRF on child’s internalizing and externalizing problems, nor on effortful control. These findings necessitate critical evaluation of the study design, especially regarding the self-reported nature of PRF.

There was some indication that our assessment of the child outcomes was influenced by common method biases. First, the mothers with high PRF may have overreported their child’s problems, as indicated by the unexpected association between prenatal high PRF and child’s higher negative affectivity during infancy. Second, the overconfident mothers may have been underreporting their child’s problem behaviors, as hinted by the exceptionally low scores on externalizing and internalizing problems among the mothers from the OHH trajectory. In general, evidence about the external validity of PRFQ is still modest (see Rutherford et al., 2013, 2015, 2018; Anis et al., 2020). Therefore, it remains unclear to what extent our positive and null results regarding the child outcomes were accounted by (a) the effects of actual PRF within the mother-infant interactions, (b) parental perceptual biases related to genuine differences in PRF, or (c) social desirability biases inherent for self-report methods. Caution must thus be used before inferring causality from our results. A conservative interpretation contextualizes our results to reflect parents’ perceptions of the child, important as such (Sameroff, 2010), rather than actual properties of the child.

It is also noteworthy to consider that we utilized infant temperament as a covariate when analyzing the child outcomes. This provided a relatively strict test of the prospective associations. To the extent the maternal PRF had genuinely shaped child’s temperament by the age of 6 months, controlling for the temperament led to underestimation of the effects of maternal PRF on the child outcomes. At the same time, controlling for the temperament may have provided some remedy against the perceptual and social desirability biases. Previous research has demonstrated that parental reports of their own child’s temperament do contain some reporter bias (Seifer et al., 2004). To the extent such reporter bias was present in our assessments of infant temperament, the bias was statistically controlled for when testing the associations between the maternal PRF and the child outcomes. Eventually, however, studies utilizing more objective and independent assessments of child development are needed to clarify this issue.

### Strengths and limitations

Our study was the first to depict the naturally occurring pathways in maternal PRF from pregnancy to child’s toddlerhood, and to analyze their associations with child outcomes. The strengths of our study involve utilizing a large general population sample, controlling for the effects of infant temperament, and involvement of both mothers and fathers reports regarding child temperament and outcomes. Yet, our study has several limitations, apart from the already discussed issues related to common method biases and causal mechanisms.

First, due to very low reliability coefficients, we had to exclude the PRFQ Prementalizing dimension from the person-oriented analyses. This limits the conceptual coverage of PRF in our study. Relatedly, the Certainty dimension differentiated only one prenatal profile (i.e., Overconfident), while in all the postnatal profiles the scores were similar. Together, these indicate that maladaptive forms of PRF may be rare in a normal population. Alternatively, the PRFQ may not be sufficiently sensitive to identify such processes among mothers of young children. Thus, it is an important area of future research to continue the psychometric work with the PRFQ. Furthermore, to identify the most meaningful PRF profiles in latent analyses, it may be beneficial to include multiple assessments of the different PRF dimensions (for an example, see Gagliardini et al., 2020).

Second, it is important to note that our sample was a general population sample. While the person-oriented analyses revealed homogenous subgroups within the data, a caution is still warranted when generalizing our results to higher risk populations. Relatedly, the most atypical and deviant PRF trajectories showed the largest associations with the child’s internalizing and externalizing problems. However, while clinically interesting, the small group sizes of these trajectories precluded their separate analyses. Future studies are needed to study the parental PRF trajectories and their associations with child outcomes in high risk and clinical populations.

Third, attrition was relatively large in our data, especially regarding the fathers. Furthermore, attrition analyses indicated that participant drop out did not occur randomly. Thus, for example, mothers with high education level and with high scores on Certainty may have been overrepresented in our study. While we used advanced statistical approach (FIML) to utilize the full data, it is still possible that the attrition introduced some biases in our study.

Fourth, we used mothers’ and fathers’ combined reports of the child temperament and developmental outcomes to minimize reporter bias. This was done on the premise that neither parent can be regarded as the “gold standard” informant of the child (De Los Reyes and Kazdin, 2005; Moens et al., 2018). Arguably, the reports could also have been treated separately as both parents have some unique information about the child. However, the number of the participating fathers was low and the resulting issues of low statistical power precluded their separate analysis. Furthermore, as the mothers were the informants of the PRF, their separate analysis would have increased rather than decreased common method biases. As such, our analyses did not fully utilize parents’ unique perspectives. In future studies, because the PRF is closely related to parent’s ways of perceiving their child, it would be beneficial to utilize also more objective assessments of the child (e.g., observations and experiments).

Finally, due to high complexity of our analyses, we did not model more sophisticated developmental moderators or mediators. For example, research suggest that there are important individual differences in susceptibility for the effects of caregiving (Belsky and van IJzendoorn, 2017). Moreover, we did not model the more specific parent (e.g., maternal-fetal attachment and sensitivity) and child (e.g., emotional and verbal abilities) processes that could mediate between parental PRF and child development. Such processes can be more effectively modeled using variable-oriented approach that focuses on the unique variability of the factors under study.

### Conclusion

Parental PRF is a core aspect of early caregiving and helps the parent to gain and sustain the child’s perspective better in mind, also under in stressful and demanding situations. As such, it is important to understand how the maternal PRF emerges and unfolds in relation to one’s own child. Our novel study contributed to previous research by depicting the naturally occurring dynamics and complexities in maternal PRF development. Our results bear both scientific and practical value, as they provide a “map” about the “landscape” the mothers travel from pregnancy to child’s toddlerhood. This knowledge can inform, for example, screening of parental problems, and perhaps more importantly, about how to develop timely and focused interventions to support smooth transition to parenthood and to strengthen the early parent–child bonding.

The PRF profiles and trajectories were validated against background variables and infant temperament, and also showed various associations with child development at the age of 2 years. These deepen our understanding about both the parent and child related preconditions and potential consequences on child development. Continuously high PRF may provide the child a predictable and sensitive environment that promotes social and emotional development. Yet, our results also raised critical questions about the nature of self-reported parental PRF and biases in mothers’ reports of their child. More research is needed to evaluate the mechanisms that explain the associations between self-reported PRF and child outcomes, and experimental intervention studies are necessary to assess the causality of such effects. Furthermore, we hope our study encourages researchers to utilize person-oriented approaches in future studies of parental mentalization.

## Data availability statement

The data analyzed in this study is subject to the following licenses/restrictions: The datasets generated for this study will not be made publicly available because of restriction imposed by the Finnish law and the study’s ethical permissions do not allow sharing of the data used in this study. Requests to access the datasets should be directed to the Principal Investigator of the FinnBrain Birth Cohort Study. Requests to access these datasets should be directed to HK (hasse.karlsson@utu.fi).

## Ethics statement

The studies involving human participants were reviewed and approved by Ethics Committee of the Hospital District of Southwest Finland (ETMK: 57/180/2011). The patients/participants provided their written informed consent to participate in this study.

## Author contributions

JL: methodology, analyses, and writing—original draft. MP: supervision, writing—review and editing, and original draft. SN: writing—review and editing. KT: data collection, and writing—review and editing. HK and LK: funding acquisition, and writing—review and editing. RK: supervision and writing—review and editing. All authors contributed to the article and approved the submitted version.

## Funding

JL was supported by the Academy of Finland (323845). MP was supported by the Academy of Finland, Hospital District of South-Western Finland, Signe and Ane Gyllenberg Foundation, Päivikki ja Sakari Sohlberg Foundation. HK was supported by the Academy of Finland (134950 and 253270), Signe and Ane Gyllenberg Foundation. RK was supported by the Signe and Ane Gyllenberg Foundation, The Academy of Finland (308252) and the Finnish Cultural Foundation.

## Conflict of interest

The authors declare that the research was conducted in the absence of any commercial or financial relationships that could be construed as a potential conflict of interest.

## Publisher’s note

All claims expressed in this article are solely those of the authors and do not necessarily represent those of their affiliated organizations, or those of the publisher, the editors and the reviewers. Any product that may be evaluated in this article, or claim that may be made by its manufacturer, is not guaranteed or endorsed by the publisher.

## Footnotes

^1^ www.finnbrain.fi
