# Supplementary material for: Early pathways of maternal mentalization: Associations with child development in the FinnBrain birth cohort study
Source: Front Psychol. 2022 Dec 5;13:855190. doi: 10.3389/fpsyg.2022.855190 (PMC9792295; doi:10.3389/fpsyg.2022.855190)
Supplement: Supplementary file 1 [file Table_1.DOCX]

Supplementary Material

# Supplementary Material 1

Dimensions, definitions and the items from P-PRFQ (Pajulo et al. 2015) used in the current study to assess mentalization at T1.

**Dimension 1: Certainty of mental states** (= being or not being aware of the limitations of knowing mental states for certain, i.e., accepting opacity)

in the current study

Item 1: As a parent, I think I will always know why my child acts the way that she/he does.

Item 2: As a parent, I think that I will always know why I act the way I do with my child.

Item 3: As a parent, I think I will always be able to predict what my child will do next.

Item 4: As a parent, I think that I will always be able to know what my child wants.

**Dimension 2: Interest and curiosity** (= reflecting the fetus-baby’s experience and perspective already prenatally, here and now)

Item 1 (reverse scored): I believe it is irrelevant at this point to try to guess what my developing baby feels.

Item 2: Now, during pregnancy, I wonder what my developing baby expects and needs from me.

Item 3: I feel that there is already a unique relationship between me and my developing baby.

Item 4: I find it fascinating to search for signs that would tell me how my developing baby is doing.

Item 5: Now, during pregnancy, I have been wondering if my developing baby may feel upset when I feel upset myself.

**Dimension 3: Dynamic nature** (= reflecting mental states in past, present and future relationships, moving fluently in time)

Item 1: Now, during pregnancy, I think about how I felt when I was a little child.

Item 2: I try to imagine which moments will be the most difficult for me with the baby after she/he is born.

Item 3: I try to imagine which moments will be the most enjoyable for me with the baby after she/he is born.

Item 4: I think about how my developing baby may be experiencing something I am doing.

Item 5: I find myself thinking of how it may have been for my mother when she was pregnant with me.

# Supplementary Material 2

Dimensions, definitions and the items from PRFQ (based on Luyten et al., 2017) used in the current study to assess mentalization at T2 and at T3

**Factor 1 Certainty about mental states, CM** (= extent of being aware of the limitations of knowing mental states for certain, i.e. accepting opacity)

Item 1: I always know what my child wants

Item 2: I can completely read my child’s mind

Item 3: I can always predict what my child will do

Item 4 (reverse scored): I can sometimes misunderstand the reactions of my child

Item 5: I always know why I do as I do with my child

Item 6: I always know why my child acts the way he or she does

**Factor 2 Interest and curiosity in mental states, IC** (= ability and willingness to reflect the child’s experience and perspective)

Item 1: I like to think about the reasons behind the way my child behaves

Item 2: I wonder a lot about what my child is thinking and feeling

Item 3: I am often curious to find out how my child feels

Item 4: I try to see situations through the eyes of my child

Item 5: I try to understand the reasons why my child misbehaves

Item 6 (reverse scored): I believe there is no point in trying to guess what my child feels

**Factor 3 Prementalizing modes, PM** (= parents’ tendency to make maladaptive, inappropriate or malevolent attributions about the child)

Item 1: The only time I’m certain my child loves me is when he or she smiles at me

Item 2: My child cries around strangers to embarrass me

Item 3: I find it hard to actively participate in make believe play with my child ⃰

Item 4: My child sometimes gets sick to keep me from doing what I want to do

Item 5: When my child is fussy he or she does that just to annoy me ⃰

Item 6: Often, my child’s behavior is too confusing to bother figuring out

## ** Excluded from the PRFQ at the child’s age of 6 months.*

# Supplementary Material 3

Table S1. Effects of the Background Variables and Infant Temperament (T2) on the Child
Outcomes at the age of 2 years (T3)

|  | Externalizing Problems | | | Internalizing Problems | | | Social-emotional competence | | | Effortful Control | | |
| --- | --- | --- | --- | --- | --- | --- | --- | --- | --- | --- | --- | --- |
|  | *B* | *SE* | *p* | *B* | *SE* | *p* | *B* | *SE* | *p* | *B* | *SE* | *p* |
| Maternal age | -0.04 | 0.03 | .127 | -0.02 | 0.03 | .575 | -0.09 | 0.03 | .000 | -0.03 | 0.03 | .349 |
| Education level | -0.06 | 0.03 | .045 | -0.06 | 0.03 | .037 | 0.13 | 0.03 | .000 | 0.06 | 0.03 | .028 |
| Parity status | -0.01 | 0.03 | .756 | 0.03 | 0.03 | .319 | 0.04 | 0.03 | .127 | 0.09 | 0.03 | .000 |
| Child's gender | -0.20 | 0.03 | .000 | 0.02 | 0.03 | .541 | 0.20 | 0.02 | .000 | 0.08 | 0.03 | .001 |
| IBQ Surgency | 0.01 | 0.03 | .683 | -0.08 | 0.03 | .006 | 0.20 | 0.03 | .000 | 0.17 | 0.03 | .000 |
| IBQ Negative affectivity | 0.16 | 0.03 | .000 | 0.29 | 0.03 | .000 | -0.06 | 0.03 | .030 | -0.11 | 0.03 | .000 |
| IBQ Regulation/ Orienting | -0.07 | 0.03 | .023 | -0.01 | 0.03 | .844 | 0.17 | 0.03 | .000 | 0.32 | 0.03 | .000 |

*Note.* The beta coefficients (B) represent standardized values. Values for parity status are 1 = primiparous, 2 = non-primiparous. Values for child’s gender are 0 = girl, 1 = boy. T2 = At the child’s age of 6 months; T3 = At the child’s age of 2 years.

# Supplementary Material 4

Table S2. Frequencies of All Separate PRF Trajectories

| Pregnancy  (T1) | Child’s age of 6 months (T2) | Child's age of 2 years (T3) | Frequency (*n*) | % | Cumulative % |
| --- | --- | --- | --- | --- | --- |
| **Average** | **High** | **High** | **1100** | **0.41** | **0.41** |
| **High** | **High** | **High** | **598** | **0.22** | **0.63** |
| **Average** | **Low** | **High** | **249** | **0.09** | **0.72** |
| **Low** | **High** | **High** | **170** | **0.06** | **0.79** |
| **Overconfident** | **High** | **High** | **137** | **0.05** | **0.84** |
| **Average** | **Low** | **Low** | **80** | **0.03** | **0.87** |
| Low | Low | High | 66 | 0.02 | 0.89 |
| High | Low | High | 64 | 0.02 | 0.92 |
| Average | High | Low | 37 | 0.01 | 0.93 |
| Low | Low | Low | 36 | 0.01 | 0.94 |
| High | High | Low | 30 | 0.01 | 0.96 |
| Average | Very low | High | 25 | 0.01 | 0.96 |
| Low | Very low | Low | 17 | 0.01 | 0.97 |
| Low | Very low | High | 16 | 0.01 | 0.98 |
| Overconfident | Low | High | 16 | 0.01 | 0.98 |
| Average | Very low | Low | 13 | 0.00 | 0.99 |
| High | Low | Low | 13 | 0.00 | 0.99 |
| Low | High | Low | 10 | 0.00 | 1.00 |
| Overconfident | Low | Low | 4 | 0.00 | 1.00 |
| High | Very low | High | 4 | 0.00 | 1.00 |
| Overconfident | High | Low | 2 | 0.00 | 1.00 |
| Overconfident | Very low | Low | 0 | 0.00 | 1.00 |
| Overconfident | Very low | High | 0 | 0.00 | 1.00 |
| High | Very low | Low | 0 | 0.00 | 1.00 |

*Note.* Bolded lines refer to the 6 most common maternal PRF trajectories (AHH, HHH, ALH, LHH, OHH, ALL) analyzed separately in the main analyses. The smaller PRF trajectories were combined to MIX group.
